# Preoperative Multi-modal Images-based Radiomics Model for Distinguishing Spinal Osteosarcoma and Chondrosarcoma

**DOI:** 10.2174/0115734056403627251022193043

**Published:** 2025-10-29

**Authors:** Chenxi Wang, Yuan Yuan, Kai Ye, Zhenyu Li, Huishu Yuan, Ning Lang

**Affiliations:** 1 Department of Radiology, Peking University Third Hospital, 49 North Garden Road, Haidian District, Beijing, 100191, People’s Republic of China; 2 Institute of Intelligent Diagnostics, Beijing United-Imaging Research Institute of Intelligent Imaging, 9 Yongteng N. Road, Beijing, 100080, People’s Republic of China; 3 Department of Radiology, State Key Laboratory of Vascular Homeostasis and Remodeling, Peking University Third Hospital, 49 North Garden Road, Haidian District, Beijing, 100191, People’s Republic of China

**Keywords:** Radiomics, Osteosarcoma, Chondrosarcoma, Differential diagnosis, Computed tomography, Magnetic resonance imaging

## Abstract

**Introduction:**

This study aimed to develop and validate a radiomics fusion model based on CT and MRI for distinguishing between spinal osteosarcoma and chondrosarcoma, and to compare the performance of models derived from different imaging modalities.

**Methods:**

A retrospective analysis was conducted on 63 patients with histologically confirmed spinal osteosarcoma (n=20) and chondrosarcoma (n=43). Radiomics features were extracted from CT and MRI (T1-weighted, T2-weighted, and T2-weighted fat-suppressed) sequences, followed by feature selection using univariate logistic regression and LASSO. Eight machine learning models were utilized to construct radiomics models, based on CT, MR, both CT and MR, and clinical information combined with CT and MR. Models were evaluated *via* five-fold cross-validation and compared against radiologists’ interpretations using the area under the receiver operating characteristic curve (AUC), accuracy, sensitivity, specificity, F1 score, and Matthews correlation coefficient.

**Results:**

The MRI-based radiomics model using linear discriminant analysis achieved the highest diagnostic performance (AUC=0.963, sensitivity=95.3%, specificity=80.0%), significantly outperforming both CT-based models (AUC=0.700) and radiologists' diagnosis (*p*<0.001). The CTMR and clinico-CTMR models did not show significant improvement over the MR model. The MR model demonstrated excellent calibration and clinical utility, with substantial net benefit across threshold probabilities.

**Discussion:**

The superior performance of the MRI-based model highlighted the value of MRI radiomics in tumor differentiation. This clinically practical tool may support preoperative diagnosis using routine MRI, potentially facilitating more timely treatment decisions.

**Conclusion:**

In conclusion, the MRI-based radiomics model enabled accurate preoperative discrimination between spinal osteosarcoma and chondrosarcoma.

## 1 INTRODUCTION

Bone tumors encompass a group of primary or secondary neoplastic lesions of the bone, characterized by diverse pathological types and biological behaviors. Osteosarcoma is the most common primary malignant bone tumor, predominantly affecting the long bones of the limbs, with spinal involvement accounting for only 4% of all primary osteosarcomas [1]. The standard treatment typically involves a combination of surgery and chemotherapy, but the prognosis remains poor, with reported long-term survival rates below 20% [2]. Furthermore, due to the complex anatomy of the spine, extensive resection is not always feasible. For patients with positive surgical margins reported on postoperative pathology, adjuvant radiotherapy can be administered to reduce the risk of local recurrence [3]. Chondrosarcoma is the third most common primary malignant bone tumor, with spinal chondrosarcomas comprising only 5% of all chondrosarcomas. The median survival for these patients is slightly over seven years [4]. The primary treatment principle for chondrosarcoma is surgical resection [5]. Given the distinct therapeutic approaches and prognoses of osteosarcoma and chondrosarcoma, reliable preoperative differentiation between these two entities is critical for selecting appropriate treatment strategies and evaluating disease progression.

Image-guided biopsy is a commonly used method for diagnosing bone tumors. However, as an invasive procedure, it carries the risk of complications, such as hematoma, neurovascular injury, infection spread, and tumor seeding along the needle tract [6]. Early diagnosis of osteosarcoma remains clinically challenging due to its potential radiographic and pathological overlap with benign lesions or low-grade malignant tumors [7]. Preoperative diagnosis primarily relies on conventional imaging modalities, including X-ray, computed tomography (CT), and magnetic resonance imaging (MRI). Although X-ray is routinely employed as the first-line imaging technique for bone tumors, CT and MRI are essential for precise evaluation of spinal lesions in this anatomically complex region [8]. CT provides critical information regarding tumor size, morphology, location, and its relationship with surrounding tissues, as well as an assessment of matrix mineralization [9]. Nevertheless, both osteosarcoma, characterized by tumor bone, and chondrosarcoma, characterized by calcification, can appear as high-density areas within the tumor on CT, making differentiation challenging. Moreover, certain histological variants of osteosarcoma may demonstrate chondroid differentiation or produce cartilaginous matrix, manifesting radiographically as “popcorn-like” calcifications or extensive fibrous tissue formation [8], which further complicates pathological classification. In contrast, MRI offers superior visualization of bone marrow involvement, soft tissue infiltration, and lesion fluid content [10].

Cè *et al*. demonstrated radiomics' broad applications in bone tumors, spanning detection, classification, and prognosis prediction (treatment response/recurrence) using various imaging modalities (X-ray, CT, MRI, SPECT) [8]. The handcrafted radiomics approach enables the extraction of quantitative features from imaging data, supporting disease diagnosis [9], pathological subtype prediction [11], and the evaluation of treatment response and prognosis [12, 13]. For instance, Nie *et al*. proposed a CT-based radiomics model for predicting the histological grade and prognosis of chondrosarcoma [13]. Similarly, Zhang *et al*. developed an MRI-based radiomics model to predict histological response to neoadjuvant chemotherapy in high-grade osteosarcoma patients, demonstrating excellent performance [12]. Pereira *et al*. introduced a machine learning-based CT radiomics model to assess the risk of pulmonary metastasis after osteosarcoma diagnosis [14].

Recent advances in multi-modal medical image fusion techniques have further enhanced the potential of radiomics analysis. Novel frameworks employing methods, such as non-subsampled shearlet transform with co-occurrence filters [15], content-aware generative adversarial networks [16], and semantic awareness joint-driven neural networks [17], have demonstrated improved performance in integrating complementary information from different imaging modalities. These approaches address key challenges in feature preservation and semantic consistency during fusion [18], providing robust foundations for developing advanced radiomics models.

While previous studies have made progress in radiomics-based differentiation of osteosarcoma and chondrosarcoma, several critical gaps remain unaddressed. Long *et al*. confirmed that integrating CT-based radiomics features with machine learning significantly enhances the diagnostic accuracy and interpretability for distinguishing osteosarcoma from chondrosarcoma [9]. While Gao *et al*. [19] performed radiomics analysis using T1CE and T2FS sequences in 106 patients with diverse skeletal involvement, their spinal and pelvis subgroup comprised only 16 cases (11 osteosarcoma and 5 chondrosarcoma). This limited sample size in both training and validation cohorts raises concerns about model generalizability for spinal lesions. Furthermore, the use of contrast-enhanced T1-weighted imaging (T1CE) introduces additional constraints, as this sequence is not routinely acquired in all clinical settings, potentially limiting the approach's widespread applicability.

Our study advances this field by specifically focusing on spinal lesions, which present distinct diagnostic challenges due to (1) their complex anatomical relationships with neural structures, (2) the low incidence of these tumors in the spine, making data acquisition particularly challenging, and (3) the need for specialized imaging protocols. We have developed a comprehensive radiomics fusion model that (1) systematically combines CT and MRI modalities to capitalize on their complementary strengths, (2) integrates clinically features aiming to improve diagnostic accuracy, and (3) provides the direct comparison of modality-specific contributions for spinal tumor differentiation. This approach addresses the critical need for spine-specific diagnostic tools while overcoming the limitations of previous studies that have either used single modalities or lacked sufficient spinal case representation.

## 2 MATERIALS AND METHODS

### 2.1 Data Acquisition

This study was approved by the Medical Science Research Ethics Committee (registration number IRB00006761-M2018251). Due to the retrospective nature of the study, the written informed consent was waived.

This single-center retrospective study included 63 patients at Peking University Third Hospital from January 2017 to November 2023.

The inclusion criteria were as follows: (1) patients having any histologically confirmed primary chondrosarcoma and primary osteosarcoma of the spine; (2) preoperative plain scan MRI and CT have been performed.

**Fig. (1):**
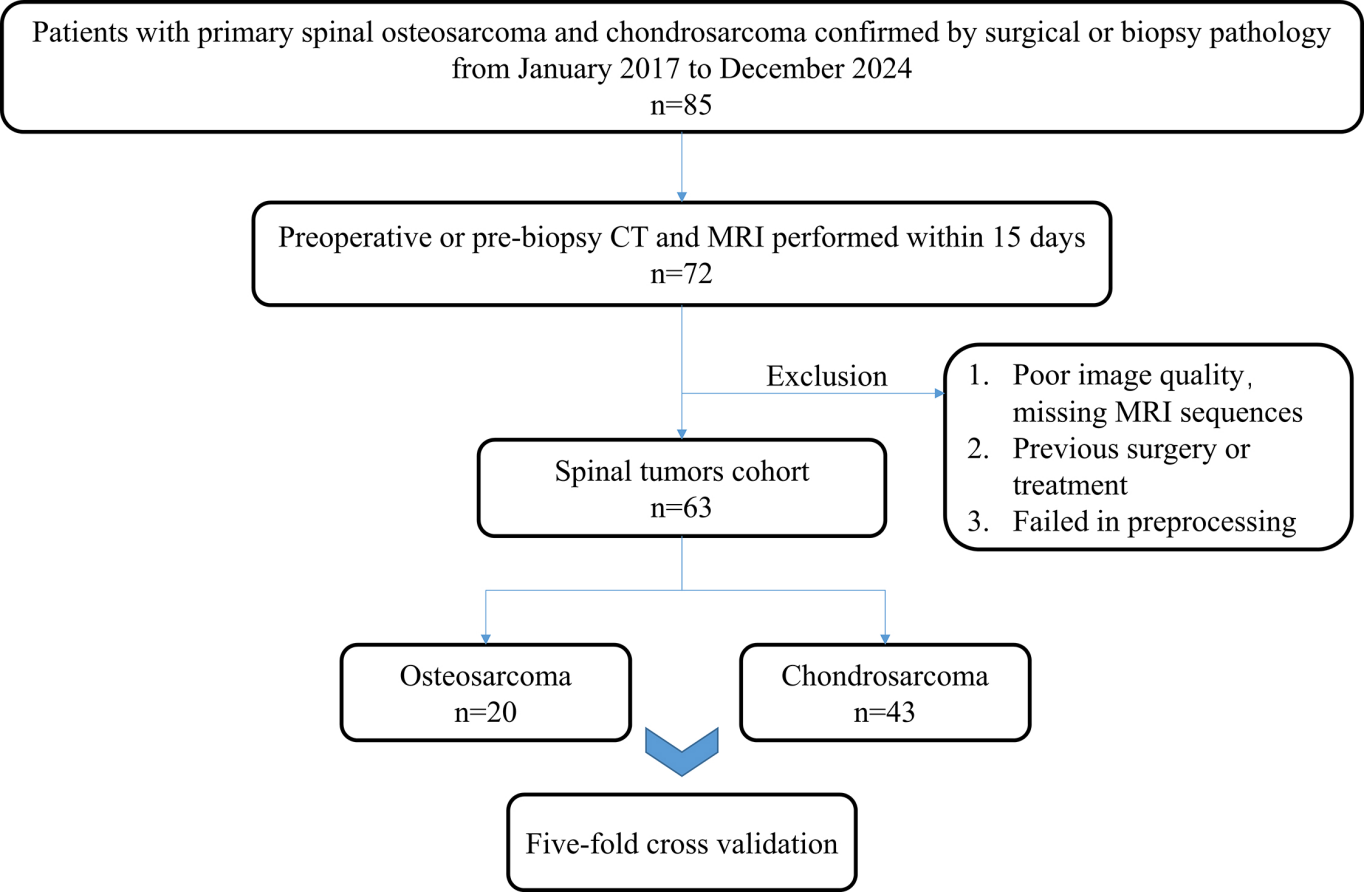
Flowchart of the inclusion and exclusion criteria.

The exclusion criteria were as follows: (1) poor image quality; (2) missing MRI sequences; (3) radiotherapy, chemotherapy, or other lesion interventions having been performed before CT and MRI imaging (Fig. **1**).

### 2.2 MRI and CT Protocol

MRI scans were carried out using either a GE Discovery MR750 3.0T scanner (GE Healthcare, Piscataway, NJ, USA) or a Siemens Magnetom Trio Tim 3.0T scanner (Siemens, Erlangen, Germany). The imaging protocol included axial, coronal, and sagittal T2-weighted imaging (T2WI), sagittal T1-weighted imaging (T1WI), and sagittal fat-suppressed T2WI (T2FS). The imaging parameters comprised a repetition time (TR) of 400-800 ms and an echo time (TE) of 10-30 ms for T1WI. For T2WI, the TR ranged from 2500 to 4000 ms and the TE from 50 to 120 ms. Gadolinium (Gadopentetate dimeglumine; Beilu Pharmaceutical, Beijing, China) was utilized as the contrast enhancement agent at a dosage of 0.2 mL/kg, administered through the elbow vein at a rate of 1 ml/s using a power injector. After injection, axial fat-suppressed T1WI scanning was performed with TR between 571 and 652 ms and TE between 9.8 and 11.2 ms.

CT imaging was conducted using either a GE Lightspeed 64-slice spiral CT (GE Medical System, Chalfont St Giles, UK) or a Siemens Somatom Definition Flash dual-source CT (Siemens, Erlangen, Germany). The imaging parameters included 120 kVp, 200–300 mAs; a collimator width of 0.625 or 0.60 mm; a pitch of 1.0; a slice thickness of 2 mm; and an interlayer distance of 3 mm.

## 3 RADIOMICS ANALYSIS TO BUILD A CLASSIFICATION MODEL

### 3.1 Tumor Segmentation on CT and MRI

**Fig. (2):**
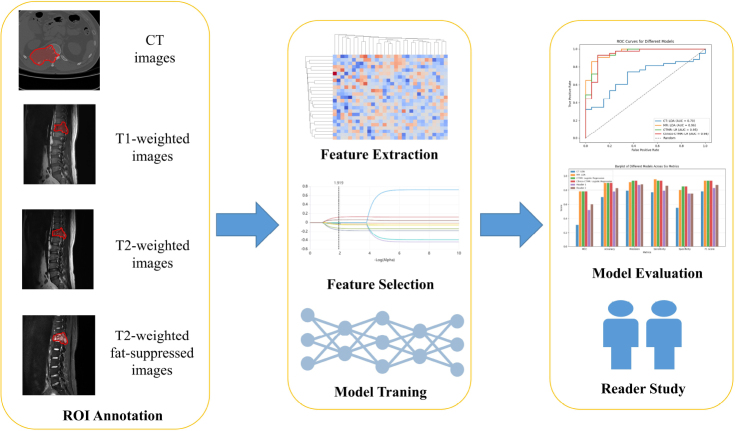
The workflow of the study. The study design encompassed tumor segmentation, radiomics feature extraction and selection, and subsequent model training and evaluation.

For each case, the lesion was located on axial CT slices, and on sagittal T1 sequences, sagittal T2 sequences, and sagittal T2FS sequences. Lesions were manually segmented using the uAI Research Portal platform 1.1 (United Imaging Intelligence, Co., Ltd.). The initial delineations were conducted by a radiologist with 2 years of experience (XX1) and validated by another with 22 years of experience (XX2). Ultimately, the regions of interest (ROIs) were obtained, including ROI_CT_, ROI_T1_, ROI_T2_, and ROI_T2FS_, respectively (Fig. **2**).

### 3.2 Feature Extraction

The medical imaging analysis followed a standardized radiomics workflow, as follows: 1) isotropic voxel resampling (1.0^3^ mm^3^) using B-spline interpolation; 2) multi-channel feature extraction *via* the uAI platform (original images + 17 filtered variants); 3) acquisition of 116 radiomic features across 8 categories covering first-order statistics, morphology, and texture patterns. Feature extraction was performed exclusively on the training data within each fold. Detailed methodologies are provided in Supplementary Appendix **S1**.

### 3.3 Feature Selection

Feature selection was performed independently for CT and each MRI sequence (T1, T2, T2FS) to account for modality-specific information encoding. The workflow consisted of two phases: univariate pre-screening and regularized feature refinement.

Logistic regression with Benjamini-Hochberg FDR correction (q<0.05) was applied to each of the 2,264 radiomic features per modality, retaining features demonstrating significant association with tumor phenotype.

Least absolute shrinkage and selection operator (LASSO) regression was implemented on the pre-screened feature subset. The optimal regularization parameter (λ=0.05) was determined through 5-fold cross-validation, maximizing the area under the receiver operating characteristic curve (AUC-ROC) while minimizing feature redundancy, resulting in the retention of 4 features per modality.

### 3.4 Machine Learning Methods

The selected 4 radiomic features per modality were normalized using Z-score normalization and utilized to construct classifiers using 8 different classification models, including AdaBoost, ExtraTrees, gradient boosted decision trees (GBDT), linear discriminant analysis (LDA), light gradient boosting machine (LGBM), logistic regression (LR), random forest (RF), and extreme gradient boosting (XGBoost). The classifiers were evaluated using five-fold cross-validation.

## 4 THE RADIOLOGISTS’ DIAGNOSIS OF THE DISEASE

Two radiologists independently evaluated the CT and MRI findings of patients without knowledge of the pathological diagnoses. Reader 1 (XX3), an attending physician specializing in musculoskeletal imaging with 10 years of experience, and reader 2 (XX4), a musculoskeletal radiologist specializing in oncology with 15 years of experience, recorded their diagnoses. The corresponding Matthews correlation coefficient (MCC), sensitivity, specificity, accuracy, and precision were calculated.

## 5 STATISTICAL ANALYSIS

All statistical analyses were performed using Python (version 3.11). For continuous variables, the Mann-Whitney U-test was employed, while the Pearson chi-square test was used for categorical variables (sex and location), with a Monte Carlo simulation (10,000 replicates) applied for the analysis of location due to small expected frequencies. The Kruskal-Wallis test was used for age. The performance of the radiomics binary classification models was evaluated using the area under the receiver operating characteristic (ROC) curve (AUC), MCC (1), accuracy (4), precision (5), sensitivity (2), specificity (3), and F1 score (6). Decision curve analysis (DCA) was performed for the radiomics models to assess the net benefit of different imaging examinations and their combinations. The 95% confidence intervals for AUC values were calculated using DeLong's non-parametric method. MCC confidence intervals were computed through bootstrap resampling (2,000 iterations). The 95% confidence intervals for accuracy, precision, sensitivity, and specificity were calculated using the Wilson method.

**Table:** 

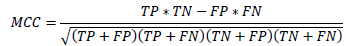	(1)

**Table:** 

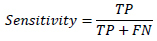	(2)

**Table:** 

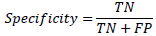	(3)

**Table:** 

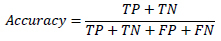	(4)

**Table:** 

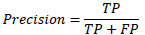	(5)

**Table:** 

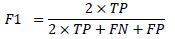	(6)

## 6 RESULTS

### 6.1 Baseline Characteristics of Patients

Based on inclusion and exclusion criteria, we enrolled 63 patients (mean age 41.8±14.2), including 32 males and 31 females. There were 20 patients (31.7%) with spinal primary osteosarcoma and 40 patients (68.3%) with spinal primary chondrosarcoma. Table **1** presents the baseline characteristics of the patients.

### 6.2 Feature Selection and Construction of the CT, MR, CTMR, and Clinico-CTMR Models

A total of 2264 radiomics features were extracted from each ROI. Univariate logistic regression combined with LASSO regression was employed for feature selection (Figs. **S1**-**S4**), with four optimal CT features retained to establish the CT radiomics model. The MR multi-modal model integrated 5 features from T1-weighted imaging, T2-weighted imaging, and T2-weighted fat-suppressed (T2FS) sequences (T1/T2/T2FS=1:1:3), demonstrating superior predictive contribution of T2FS features through hierarchical weighting analysis. The CTMR fusion model combined CT and MR features to construct a 7-dimensional feature space (CT/T1/T2/T2FS=2:1:1:3). The clinical-CTMR integrated model further incorporated clinical parameters (sex, age) with radiomics features. All models were developed using 5-fold cross-validation and eight machine learning algorithms.

### 6.3 Performance Comparison of the CT, MR, CTMR, and Clinico-CTMR Models

**Fig. (3):**
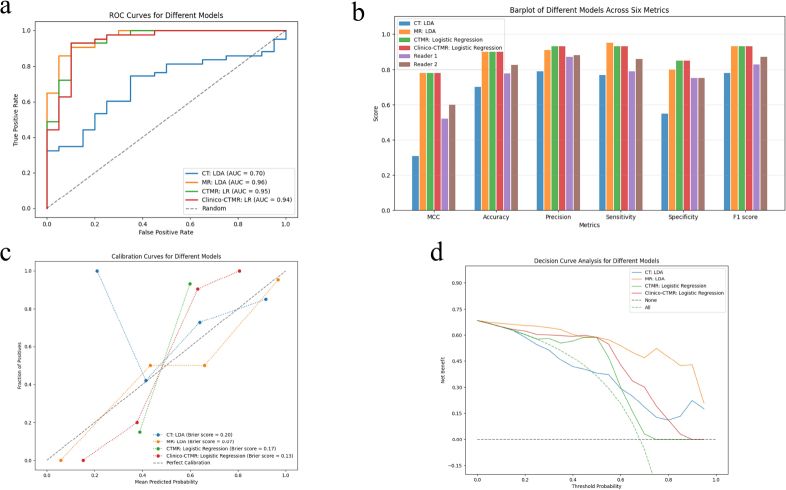
Comparative diagnostic performance between machine learning models and clinical readers. (**a**) Receiver operating characteristic (ROC) curves for the CT, MR, CTMR, and clinico-CTMR models. X-axis: False positive rate (1-specificity); Y-axis: True positive rate (sensitivity). Random guessing (dashed line, AUC=0.5). (**b**) Diagnostic performance across six classification metrics of CT, MR, CTMR, clinico-CTMR models, and clinical readers. X-axis: Evaluation metrics, including MCC, accuracy, precision, sensitivity, specificity, and F1 score. Y-axis: Score values ranging from 0.0 to 1.0. (**c**) Calibration curves for the CT, MR, CTMR, and clinico-CTMR models. X-axis: Mean predicted probability (binned from 0.0 to 1.0). Y-axis: Observed event frequency. Perfect calibration reference (dashed line). Lower Brier scores indicate better calibration. (**d**) Decision curve analysis for the CT, MR, CTMR, and clinico-CTMR models. X-axis: Probability threshold for clinical intervention (0.0-1.0). Y-axis: Net benefit accounting for true positives and false positives. “Treat none” (gray dashed line) and “treat all” (green dashed line). Models shown: CT with LDA (blue), MR with LDA (orange), CT+MR with LR (green), clinical+CT+MR with LR (red), reader 1 (purple), and reader 2 (brown). LDA, Linear discriminant analysis; LR, Logistic regression; MCC, Matthews correlation coefficient.

The eight evaluated machine learning algorithms demonstrated robust diagnostic performance across MR-, CTMR-, and clinico-CTMR-based models (Tables **2**, **S1**-**S4**; Fig. (**3**)). Notably, linear discriminant analysis (LDA) achieved exceptional performance in the MR model, with an area under the receiver operating characteristic curve (AUC) of 0.963 (95% CI: 0.939-0.986) (Fig. **3a**). This model also exhibited superior classification balance, as evidenced by a Matthews correlation coefficient (MCC) of 0.776 (95% CI: 0.598-0.929) and an F1-score of 0.932. Intriguingly, neither CTMR (*p*=1.00) nor clinico-CTMR (*p*=1.00) models demonstrated significant diagnostic improvement over the MR model through feature integration. In CT-based models, LDA remained the top-performing algorithm (AUC=0.700, 95% CI: 0.681-0.719), though its overall diagnostic efficacy was significantly inferior to MR models (*p*=0.007).

The MR-based radiomics model outperformed independent interpretations by radiologists across multiple metrics (Table **2**, Fig. (**3b**)). The model demonstrated 95.3% sensitivity (*vs*. 79.1% and 86.0% for readers) and 80.0% specificity (*vs*. 75.0% for both readers). Comprehensive evaluation revealed statistically superior performance in MCC (0.776 *vs*. 0.518 and 0.603), accuracy (0.905 *vs*. 0.778 and 0.825), precision (0.911 *vs*. 0.872 and 0.881), and F1-score (0.932 *vs*. 0.829 and 0.871), with all comparisons reaching statistical significance (*p*<0.001).

Calibration curve analysis revealed excellent agreement between predicted probabilities and observed outcomes (Brier score=0.07), with the curve closely approximating the ideal diagonal (Fig. **3c**). Decision curve analysis demonstrated consistent net clinical benefit across all threshold probabilities when utilizing the MR model, as shown in Fig. (**3d**), supporting its potential for real-world clinical implementation. To provide a comprehensive comparison of model performance across multiple metrics, radar charts (Figs. **S5**-**S6**) were added to holistically compare model performance across all six metrics.

### 6.4 Performance of the Radiomics Models in Controversial Cases

As can be seen in Fig. (**4a** and **b**), both radiologists misdiagnosed osteosarcoma as chondrosarcoma. In case A, CT images showed multiple nodular and ring-like high-density shadows. On MRI T2-weighted sequences, although most areas exhibited low signal intensity, the non-calcified regions showed high signal intensity, leading to a misdiagnosis of chondrosarcoma. In case B, CT images revealed osteolytic bone destruction with a scalloped indentation at the transition zone of the vertebral body, sclerotic margins, and small patchy high-density shadows at the posterior edge. On MRI T2-weighted sequences, the lesion predominantly showed low signal intensity with some areas of high signal intensity, resulting in a misdiagnosis of chondrosarcoma.

As can be found in Fig. (**4c** and **d**), the radiologists misdiagnosed chondrosarcoma as osteosarcoma. In case C, CT images displayed patchy and cloud-like high-density shadows within the lesion. On MRI T2-weighted images, the soft tissue mass exhibited slightly low signal intensity, leading a less experienced radiologist to misdiagnose it as osteosarcoma. In case D, CT images showed ill-defined lesion boundaries without scalloped or lobulated indentations at the junction with normal bone, and the vertebral body exhibited an ivory-like appearance. On MRI, the soft tissue mass showed slightly high signal intensity, prompting both radiologists to misdiagnose it as osteosarcoma. In contrast, the MR radiomics model consistently provided accurate diagnoses, demonstrating its ability to capture deep-seated features of lesions and achieve precise differentiation.

## 7 DISCUSSION

This study has investigated the effectiveness of radiomics models combining CT and MRI sequences for distinguishing spinal osteosarcoma and chondrosarcoma. The results revealed that the MRI fusion model integrating multiple sequences demonstrated superior performance, outperforming both the CTMR feature fusion model and the clinico-CTMR integrated model. The diagnostic performance of the MR model, in terms of AUC, sensitivity, and specificity, significantly surpassed that of both junior and senior radiologists. Notably, the CT model showed the lowest diagnostic efficacy among all evaluated approaches. The MRI fusion model exhibited optimal calibration performance and generated substantial net benefit, suggesting its potential as a reliable decision-support tool for clinicians to develop personalized treatment strategies. These findings emphasize the diagnostic value of comprehensive MRI sequence analysis over multi-modal CTMR integration in this clinical context.

Compared to previous studies, this research study has some methodological advantages. Long *et al*. have demonstrated the effectiveness of combining CT-based machine learning and radiomic features in improving the diagnostic accuracy and interpretability for osteosarcoma and chondrosarcoma [9]. Our study integrated data from different imaging modalities, including clinical information, using a multi-modal approach, thereby enhancing both diagnostic accuracy and comprehensiveness. Gao *et al*. [19] employed MRI radiomics (T1CE+T2FS), but their spinal subgroup contained only 16 cases (11 osteosarcoma and 5 chondrosarcoma). Their reliance on contrast-enhanced sequences (T1CE) further limited clinical applicability, a limitation we overcame by using routine non-contrast protocols.

CT plays a crucial role in evaluating matrix mineralization in bone tumors, particularly in the assessment of chondroid and cartilage matrix mineralization, and it is superior to other imaging modalities in visualizing calcifications within tumors [20, 21]. For instance, the characteristic CT feature of osteosarcoma is intratumoral ossification, while chondrosarcoma typically exhibits ring or arc-shaped calcifications, which are critical for diagnosing cartilage-originating tumors. However, atypical chondrosarcomas may also present with irregular patchy or punctate calcifications. Additionally, CT is especially valuable in assessing lesions in complex anatomical structures and is effective in visualizing periosteal reactions, which are strongly associated with malignant tumors [22, 23].

In contrast, MRI outperforms CT in delineating the local extent of tumors, particularly in evaluating spinal canal and soft tissue involvement. MRI also provides significant value in visualizing soft tissue masses, which is essential for assessing the invasive potential of tumors [21, 23]. T2FS images are sensitive to water tissue content and can be used to estimate cellular density [24]. This likely provided more discriminative spatial and textural features for differentiating osteosarcoma and chondrosarcoma, particularly given their overlapping mineralization patterns on CT. Furthermore, potential information redundancy between modalities, where MRI captures critical diagnostic features, such as soft tissue invasion and tumor extent, may have diminished the incremental value of CT-derived mineralization data, while introducing non-discriminative noise through multi-modal feature fusion. This may explain why the CTMR model did not outperform the MR-based model.

The increased complexity of multi-modal models (CTMR and clinico-CTMR models) may lead to overfitting, particularly with limited sample sizes, as additional modalities can introduce redundant or noisy features without providing sufficient incremental diagnostic value. This likely explains their inferior performance compared to the MR-based model, which benefits from a more focused feature space and reduced dimensionality, enhancing its diagnostic reliability in distinguishing osteosarcoma from chondrosarcoma.

According to the SEER database, the mean age of onset for spinal osteosarcoma is 45.2 years, with females accounting for approximately 46.9% of cases [25], while the mean age of onset for spinal chondrosarcoma is 51.6 years, with females constituting about 37.3% of cases [26]. Although we incorporated age and sex to explore their diagnostic potential, their limited discriminatory power in differentiating osteosarcoma from chondrosarcoma resulted in insufficient complementary information, failing to significantly enhance model performance. This suggests that clinical variables alone may not provide meaningful diagnostic value in this context.

Radiomics extracts and analyzes high-dimensional features from medical images to reveal disease characteristics [9]. In this study, we employed eight machine learning models for classification. LDA and LR achieved the best performance across modalities. LDA optimizes feature projection by maximizing inter-class differences and minimizing intra-class differences, making it suitable for tasks with distinct class separability. LR directly optimizes classification through probabilistic modeling, offering simplicity and stability for small datasets. As linear models, LDA and LR exhibit low computational complexity and strong resistance to overfitting, which is critical given the small sample size (63 cases) and imbalanced distribution (20 *vs*. 43 cases) in this study. Complex ensemble methods (*e.g*., RF, XGBoost) may underperform due to overfitting in such scenarios. Additionally, LDA and LR are robust to feature scaling and noise, aligning well with standardized radiomics features.

## 8 STUDY LIMITATIONS

This study involved several limitations. First, it was a single-center retrospective study. Primary malignant bone tumors of the spine are rare, accounting for less than 5% of all bone tumors [27], with spinal osteosarcoma comprising only 3-5% of primary malignant spinal bone tumors [28], and spinal chondrosarcoma accounting for approximately 26% [4]. As a result, only 64 patients were included in this study. Further validation of the model’s generalizability is needed in multicenter, prospective datasets. Second, despite applying strict inclusion and exclusion criteria, potential selection bias could not be entirely ruled out. We only included cases of spinal osteosarcoma and chondrosarcoma confirmed by histopathology, excluding clinically diagnosed cases. Third, this study did not explore the interpretability and visualization of the model. Future work should aim to establish associations between radiomic features and the biological characteristics of tumors and employ visualization techniques, such as Grad-CAM, to better understand the model’s prediction process.

Beyond the current limitations, this study holds promise for clinical translation. The developed MRI-based radiomics model offers immediate practical utility as a decision-support tool for ambiguous spinal tumor cases, particularly in centers lacking specialized musculoskeletal tumor expertise. Its superior performance to human readers (AUC 0.923 *vs*. radiologists' 0.716-0.792) demonstrates its potential to serve as a valuable adjunct to clinical decision-making. The findings establish an important foundation for future research directions, including multicenter validation studies and real-time clinical integration through PACS-embedded solutions. The successful application of radiomics in these rare tumors also serves as a valuable template for extending similar approaches to other uncommon spinal pathologies where traditional diagnostic paradigms remain inadequate.

## CONCLUSION

In conclusion, the proposed radiomics fusion model based on MRI demonstrated high accuracy in distinguishing between spinal osteosarcoma and chondrosarcoma, surpassing the performance of clinical experts. Therefore, our model has the potential to facilitate personalized treatment for patients with spinal osteosarcoma and chondrosarcoma, contributing to the advancement of precision medicine.

## Figures and Tables

**Fig. (4):**
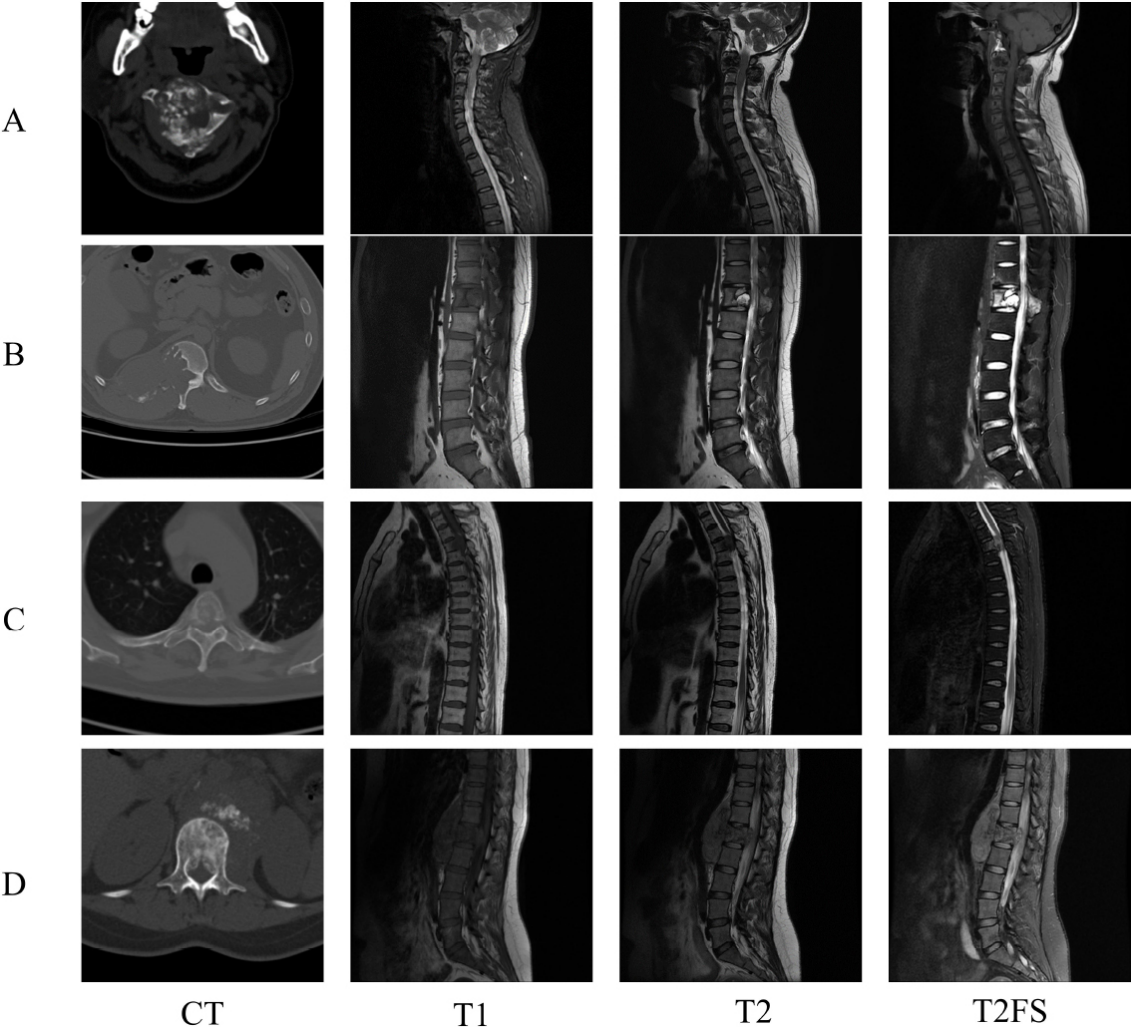
Controversial cases of osteosarcoma and chondrosarcoma. **a**, **b**: Cases where radiologists misdiagnosed osteosarcoma as chondrosarcoma; **c**, **d**: Cases where radiologists misdiagnosed chondrosarcoma as osteosarcoma. Notably, the radiomics model achieved correct diagnoses in all cases. T2FS, T2-weighted fat-suppressed.

**Table 1:** Baseline characteristics of the patients.

Variable	All Samples	Osteosarcoma	Chondrosarcoma	*p*-value
Included patients	63	20	43	-
Age, y	41.8±14.2	41.5±14.3	41.9±14.3	1.00
Men (n,%)	32/63 (50.8%)	12/20 (60.0%)	20/43 (46.5%)	0.47
Location	-	-	-	-
Cervical	24 (38.1%)	9 (45.0%)	15 (34.9%)	0.98
Thoracic	24 (38.1%)	6 (30.0%)	18 (41.9%)	-
Lumbar	12 (19.0%)	5 (25.0%)	7 (16.3%)	-
Sacrococcygeal	3 (4.8%)	0 (0.0%)	3 (7.0%)	-

**Note: **Data are expressed as mean ± standard deviation or as count with percentage in parentheses. Statistical significance of differences between osteosarcoma and chondrosarcoma was estimated using Pearson’s chi-square test for sex and location, with Monte Carlo simulation (10,000 replicates) applied for the analysis of location due to small expected frequencies. The Kruskal-Wallis test was used for age.

**Table 2:** Prediction performance of radiologists and radiomics models.

-	AUC (95%CI)	MCC	Accuracy	Precision	Sensitivity	Specificity	F1 Score
CT: LDA	0.700 (0.681,0.719)	0.313 (0.059,0.565)	0.698 (0.576,0.798)	0.786 (0.641,0.883)	0.767 (0.623,0.868)	0.550 (0.342,0.742)	0.776
MR: LDA	0.963 (0.939,0.986)	0.776 (0.598,0.929)	0.905 (0.807,0.956)	0.911 (0.793,0.965)	0.953 (0.845,0.987)	0.800 (0.584,0.919)	0.932
CTMR: LR	0.948 (0.940,0.955)	0.780 (0.596,0.930)	0.905 (0.807,0.956)	0.930 (0.814,0.976)	0.930 (0.814,0.976)	0.850 (0.640,0.948)	0.930
Clinico-CTMR: LR	0.940 (0.927,0.952)	0.780 (0.598,0.930)	0.905 (0.807,0.956)	0.930 (0.814,0.976)	0.930 (0.814,0.976)	0.850 (0.640,0.948)	0.930
Reader 1	-	0.518 (0.288,0.716)	0.778 (0.661,0.863)	0.872 (0.733,0.944)	0.791 (0.648,0.886)	0.750 (0.531,0.888)	0.829
Reader 2	-	0.603 (0.384,0.798)	0.825 (0.714,0.900)	0.881 (0.750,0.948)	0.860 (0.727,0.934)	0.750 (0.531,0.888)	0.871

**Note: **AUC, Area under the receiver operating characteristic curve; CI, Confidence interval; MCC, Matthews correlation coefficient. The 95% confidence intervals for AUC values were calculated using DeLong's non-parametric method. Confidence intervals for accuracy, precision, sensitivity, and specificity were derived using the Wilson score method and presented as percentage estimates within square brackets. MCC confidence intervals were computed through bootstrap resampling (2,000 iterations) and reported in parentheses.

## Data Availability

The data and supportive information are available within the article.

## LIST OF ABBREVIATIONS

GBDT: Gradient boosted decision trees
LDA: Linear discriminant analysis
LGBM: Light gradient boosting machine
LR: Logistic regression
RF: Random forest
XGBoost: Extreme gradient boosting
MCC: Matthews correlation coefficient
ROC: Receiver operating characteristic curve
AUC: Area under the receiver operating characteristic curve

## STANDARDS OF REPORTING

STROBE guidelines were followed.
