# Supplementary material for: Preoperative Multi-modal Images-based Radiomics Model for Distinguishing Spinal Osteosarcoma and Chondrosarcoma
Source: Curr Med Imaging. 2025 Oct 29;21:e15734056403627. doi: 10.2174/0115734056403627251022193043 (PMC13137359; doi:10.2174/0115734056403627251022193043)
Supplement: Supplementary file 1 [file CMIM-21-E15734056403627_SD1.pdf]

1573-4056/25 *Send Orders for Reprints to reprints@benthamscience.net*

BENTHAM SCIENCE

CrossMark

Current Medical Imaging

Content list available at: https://benthamscience.com/journals/cmir

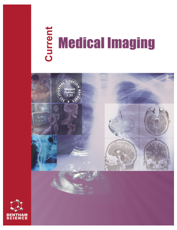

Supplementary Material

Preoperative Multi-model Images-based Radiomics Model for Distinguishing Spinal Osteosarcoma and Chondrosarcoma

Chenxi Wang[1,#], Yuan Yuan[1,#], Kai Ye[1], Zhenyu Li[2], Huishu Yuan[1] and Ning Lang[3,*]

[1]*Department of Radiology, Peking University Third Hospital, 49 North Garden Road, Haidian District, Beijing, 100191, People's Republic of China*
[2]*Institute of Intelligent Diagnostics, Beijing United-Imaging Research Institute of Intelligent Imaging, 9 Yongteng N. Road, Beijing, 100080, People's Republic of China*
[3]*Department of Radiology, State Key Laboratory of Vascular Homeostasis and Remodeling, Peking University Third Hospital, 49 North Garden Road, Haidian District, Beijing, 100191, People's Republic of China*

Appendix S1

The B-Spline interpolation method was used for resampling, resulting in all images being resampled to have an isotropic voxel spacing of 1.0 × 1.0 × 1.0 mm. Feature extraction was conducted on the uAI Research Portal platform 1.1. Extract original features and filtered features from both the original and filtered images. The filtered images are generated by using 17 types of filters, including BoxMean, AdditiveGaussiannoise, BinomialBlurImage, CurvatureFlow, BoxSigmaImage, LoG, Wavelet, Normalize, LaplacianSharpening, DiscreteGaussian, Mean, Median, SpeckleNoise, Recursive Gaussian, ShotNoise, Bilateral, SmoothingRecursiveGaussian. The extracted radiomic features include the following 8 major categories: (1) first order features (19 features); (2) 2D shape features; (3) 3D shape features; (4) Gray Level Co-occurrence Matrix (GLCM) features (24 features); (5) Gray Level Size Zone Matrix (GLSZM) features (16 features); (6) Gray Level Run Length Matrix (GLRLM) features (16 features); (7) Neighboring Gray Tone Difference Matrix (NGTDM) features (5 features); (8) Gray Level Dependence Matrix (GLDM) features (14 features).

Table S1. Prediction performance of CT-based radiomics models.

-	AUC (95%CI)	MCC	Accuracy	Precision	Sensitivity	Specificity	F1 Score
Adaboost	0.551 (0.547,0.555)	0.022 (-0.224,0.285)	0.587 (0.464,0.700)	0.689 (0.543,0.805)	0.721 (0.573,0.833)	0.300 (0.145,0.519)	0.705
ExtraTrees	0.445 (0.441,0.450)	0.000 (0.000,0.000)	0.683 (0.560,0.784)	0.683 (0.560,0.784)	1.000 (0.918,1.000)	0.000 (0.000,0.161)	0.811
GBDT	0.329 (0.320,0.338)	-0.152 (-0.246,0.000)	0.635 (0.511,0.743)	0.667 (0.541,0.773)	0.930 (0.814,0.976)	0.000 (0.000,0.161)	0.777
**LDA**	**0.700 (0.681,0.719)**	**0.313 (0.059,0.565)**	**0.698 (0.576,0.798)**	**0.786 (0.641,0.883)**	**0.767 (0.623,0.868)**	**0.550 (0.342,0.742)**	**0.776**
LightGBM	0.593 (0.581,0.605)	-0.024 (-0.241,0.240)	0.635 (0.511,0.743)	0.679 (0.548,0.786)	0.884 (0.755,0.949)	0.100 (0.028, 0.301)	0.768
Logistic Regression	0.426 (0.405,0.446)	0.000 (0.000,0.000)	0.683 (0.560,0.784)	0.683 (0.560,0.784)	1.000 (0.918,1.000)	0.000 (0.000,0.161)	0.811
Random Forest	0.426 (0.405,0.446)	-0.141 (-0.354,0.106)	0.540 (0.418,0.657)	0.646 (0.504,0.766)	0.721 (0.573,0.833)	0.150 (0.052, 0.360)	0.681
XGBoost	0.484 (0.465,0.503)	0.135 (-0.125,0.395)	0.667 (0.544,0.771)	0.712 (0.577,0.817)	0.860 (0.727,0.934)	0.250 (0.112, 0.469)	0.779

Note: AUC, area under the receiver operating characteristic curve; CI, confidence interval; MCC, Matthews correlation coefficient. The 95% confidence intervals for AUC values were calculated using DeLong's non-parametric method. Confidence intervals for accuracy, precision, sensitivity, and specificity were derived using the Wilson score method and presented as percentage estimates within square brackets. MCC confidence intervals were computed through bootstrap resampling (2,000 iterations) and reported in parentheses.

DOI: 10.2174/0115734056403627251022193043, 2025, *21*, e15734056403627

Table S2. Prediction performance of MR-based radiomics models.

-	AUC (95%CI)	MCC	Accuracy	Precision	Sensitivity	Specificity	F1 Score
Adaboost	0.910 (0.893,0.928)	0.780 (0.607,0.929)	0.905 (0.807,0.956)	0.930 (0.814,0.976)	0.930 (0.814,0.976)	0.850 (0.640,0.948)	0.930
ExtraTrees	0.953 (0.935,0.972)	0.741 (0.534,0.902)	0.889 (0.788,0.945)	0.909 (0.788,0.964)	0.930 (0.814,0.976)	0.800 (0.584,0.919)	0.920
GBDT	0.937 (0.927,0.948)	0.702 (0.503,0.867)	0.873 (0.769,0.934)	0.857 (0.733,0.929)	0.977 (0.879,0.996)	0.650 (0.433,0.819)	0.913
**LDA**	**0.963 (0.939,0.986)**	**0.776 (0.598,0.929)**	**0.905 (0.807,0.956)**	**0.911 (0.793,0.965)**	**0.953 (0.845,0.987)**	**0.800 (0.584,0.919)**	**0.932**
LightGBM	0.912 (0.884,0.940)	0.666 (0.445,0.853)	0.857 (0.750,0.923)	0.886 (0.760,0.950)	0.907 (0.784,0.963)	0.750 (0.531, 0.888)	0.897
Logistic Regression	0.952 (0.933,0.972)	0.738 (0.535,0.906)	0.889 (0.788,0.945)	0.891 (0.770,0.953)	0.953 (0.845,0.987)	0.750 (0.531, 0.888)	0.921
Random Forest	0.958 (0.939,0.978)	0.701 (0.491,0.879)	0.873 (0.769,0.934)	0.889 (0.765,0.952)	0.930 (0.814,0.976)	0.750 (0.531, 0.888)	0.909
XGBoost	0.921 (0.900,0.942)	0.701 (0.489,0.888)	0.873 (0.769,0.934)	0.889 (0.765,0.952)	0.930 (0.814,0.976)	0.750 (0.531, 0.888)	0.909

Note: AUC, area under the receiver operating characteristic curve; CI, confidence interval; MCC, Matthews correlation coefficient. The 95% confidence intervals for AUC values were calculated using DeLong's non-parametric method. Confidence intervals for accuracy, precision, sensitivity, and specificity were derived using the Wilson score method and presented as percentage estimates within square brackets. MCC confidence intervals were computed through bootstrap resampling (2,000 iterations) and reported in parentheses.

Table S3. Prediction performance of CTMR-based radiomics models.

-	AUC (95%CI)	MCC	Accuracy	Precision	Sensitivity	Specificity	F1 Score
Adaboost	0.892 (0.874,0.910)	0.741 (0.541,0.912)	0.889 (0.788,0.945)	0.909 (0.788,0.964)	0.930 (0.814,0.976)	0.800 (0.584,0.919)	0.920
ExtraTrees	0.937 (0.923,0.951)	0.660 (0.450,0.845)	0.857 (0.750,0.923)	0.854 (0.728,0.928)	0.953 (0.845,0.987)	0.650 (0.433,0.819)	0.901
GBDT	0.927 (0.917,0.937)	0.702 (0.513,0.868)	0.873 (0.769,0.934)	0.857 (0.733,0.929)	0.977 (0.879,0.996)	0.650 (0.433,0.819)	0.913
LDA	0.937 (0.913,0.962)	0.738 (0.536,0.908)	0.889 (0.788,0.945)	0.891 (0.770,0.953)	0.953 (0.845,0.987)	0.750 (0.531,0.888)	0.921
LightGBM	0.888 (0.860,0.916)	0.666 (0.447,0.856)	0.857 (0.750,0.923)	0.886 (0.760,0.950)	0.907 (0.784,0.963)	0.750 (0.531,0.888)	0.897
**Logistic Regression**	**0.948 (0.940,0.955)**	**0.780 (0.596,0.930)**	**0.905 (0.807,0.956)**	**0.930 (0.814,0.976)**	0.930 (0.814,0.976)	**0.850 (0.640,0.948)**	**0.930**
Random Forest	0.944 (0.926,0.963)	0.621 (0.405,0.820)	0.841 (0.732,0.911)	0.851 (0.723,0.926)	0.930 (0.814,0.976)	0.650 (0.433,0.819)	0.889
XGBoost	0.899 (0.874,0.923)	0.661 (0.438,0.852)	0.857 (0.750,0.923)	0.870 (0.743,0.939)	0.930 (0.814,0.976)	0.700 (0.481,0.855)	0.899

Note: AUC, area under the receiver operating characteristic curve; CI, confidence interval; MCC, Matthews correlation coefficient. The 95% confidence intervals for AUC values were calculated using DeLong's non-parametric method. Confidence intervals for accuracy, precision, sensitivity, and specificity were derived using the Wilson score method and presented as percentage estimates within square brackets. MCC confidence intervals were computed through bootstrap resampling (2,000 iterations) and reported in parentheses.

Table S4. Prediction performance of Clinico-CTMR-based radiomics models.

-	AUC (95%CI)	MCC	Accuracy	Precision	Sensitivity	Specificity	F1 Score
Adaboost	0.890 (0.871,0.909)	0.661 (0.451,0.852)	0.857 (0.750,0.923)	0.870 (0.743,0.939)	0.930 (0.814,0.976)	0.700 (0.481,0.855)	0.899
ExtraTrees	0.934 (0.918,0.949)	0.621 (0.385,0.815)	0.841 (0.732,0.911)	0.851 (0.723,0.926)	0.930 (0.814,0.976)	0.650 (0.433,0.819)	0.889
GBDT	0.909 (0.874,0.945)	0.584 (0.357,0.797)	0.825 (0.714,0.900)	0.848 (0.718,0.924)	0.907 (0.784,0.963)	0.650 (0.433,0.819)	0.876
LDA	0.936 (0.910,0.962)	0.777 (0.603,0.929)	0.905 (0.807,0.956)	0.894 (0.774,0.954)	0.977 (0.879,0.996)	0.750 (0.531,0.888)	0.933
LightGBM	0.845 (0.814,0.877)	0.545 (0.315,0.753)	0.794 (0.678,0.875)	0.875 (0.739,0.945)	0.814 (0.674,0.903)	0.750 (0.531,0.888)	0.843
**Logistic Regression**	**0.940 (0.927,0.952)**	**0.780 (0.598,0.930)**	**0.905 (0.807,0.956)**	**0.930 (0.814,0.976)**	**0.930 (0.814,0.976)**	**0.850 (0.640,0.948)**	**0.930**
Random Forest	0.938 (0.921,0.956)	0.741 (0.550,0.899)	0.889 (0.788,0.945)	0.909 (0.788,0.964)	0.930 (0.814,0.976)	0.800 (0.584,0.919)	0.920
XGBoost	0.900 (0.877,0.923)	0.660 (0.440,0.842)	0.857 (0.750,0.923)	0.854 (0.728,0.928)	0.953 (0.845,0.987)	0.650 (0.433,0.819)	0.901

Note: AUC, area under the receiver operating characteristic curve; CI, confidence interval; MCC, Matthews correlation coefficient. The 95% confidence intervals for AUC values were calculated using DeLong's non-parametric method. Confidence intervals for accuracy, precision, sensitivity, and specificity were derived using the Wilson score method and presented as percentage estimates within square brackets. MCC confidence intervals were computed through bootstrap resampling (2,000 iterations) and reported in parentheses.

Table S5. Model sizes and the inference time.

Model	Size (kb)	Time (ms)
CT: linear discriminant analysis	1.16	0.2074 ± 0.3341
MR: linear discriminant analysis	1.02	0.1109 ± 0.1518
CTMR: logistic regression	0.76	0.1229 ± 0.1328
Clinico-CTMR: logistic regression	0.77	0.1154 ± 0.0598

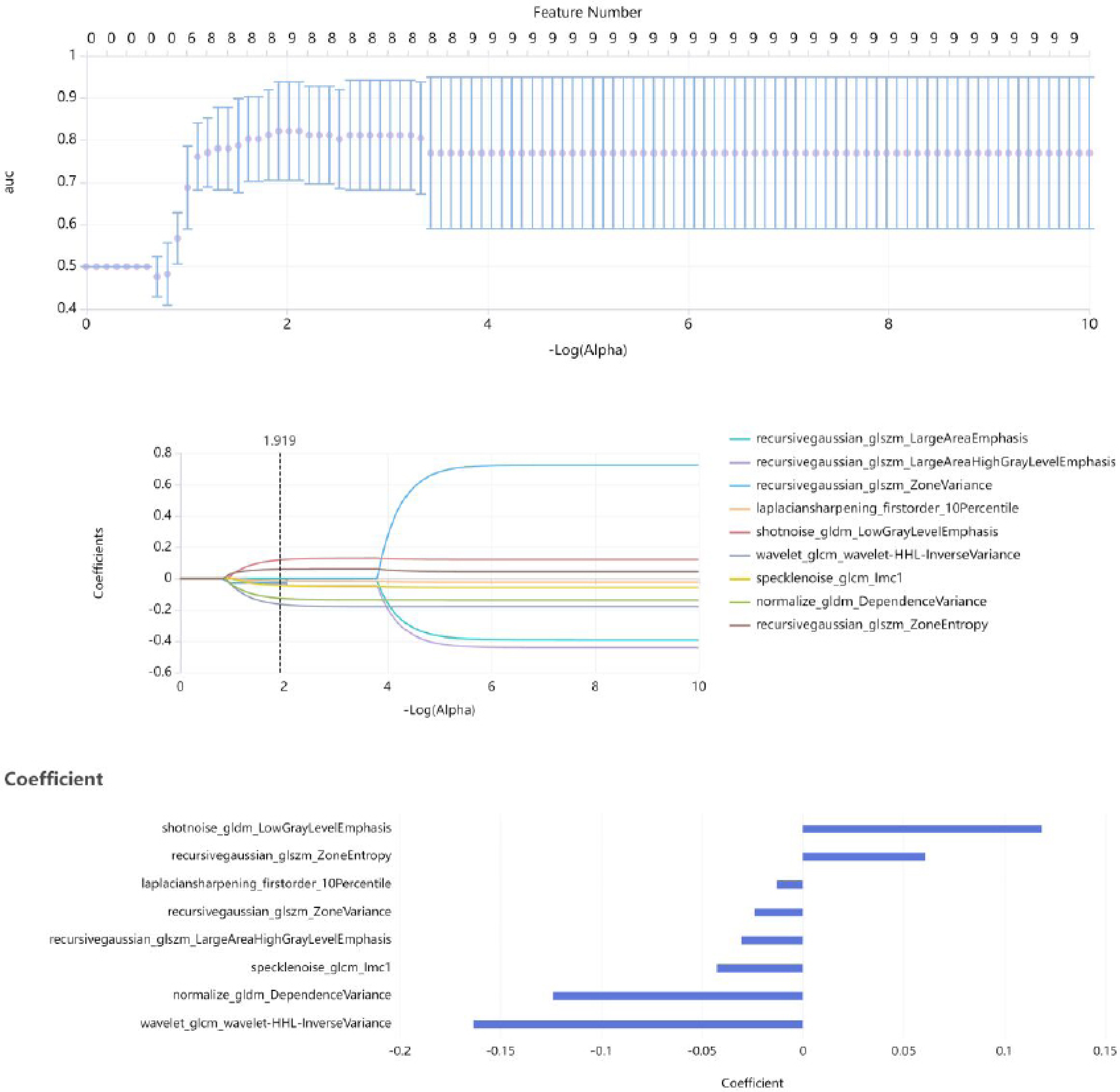

Fig. (S1). Radiomics feature extraction from CT regions of interest.
The feature selection process involves preliminary elimination of irrelevant features through univariate analysis, followed by refined multivariate selection using Least Absolute Shrinkage and Selection Operator (LASSO) regression.

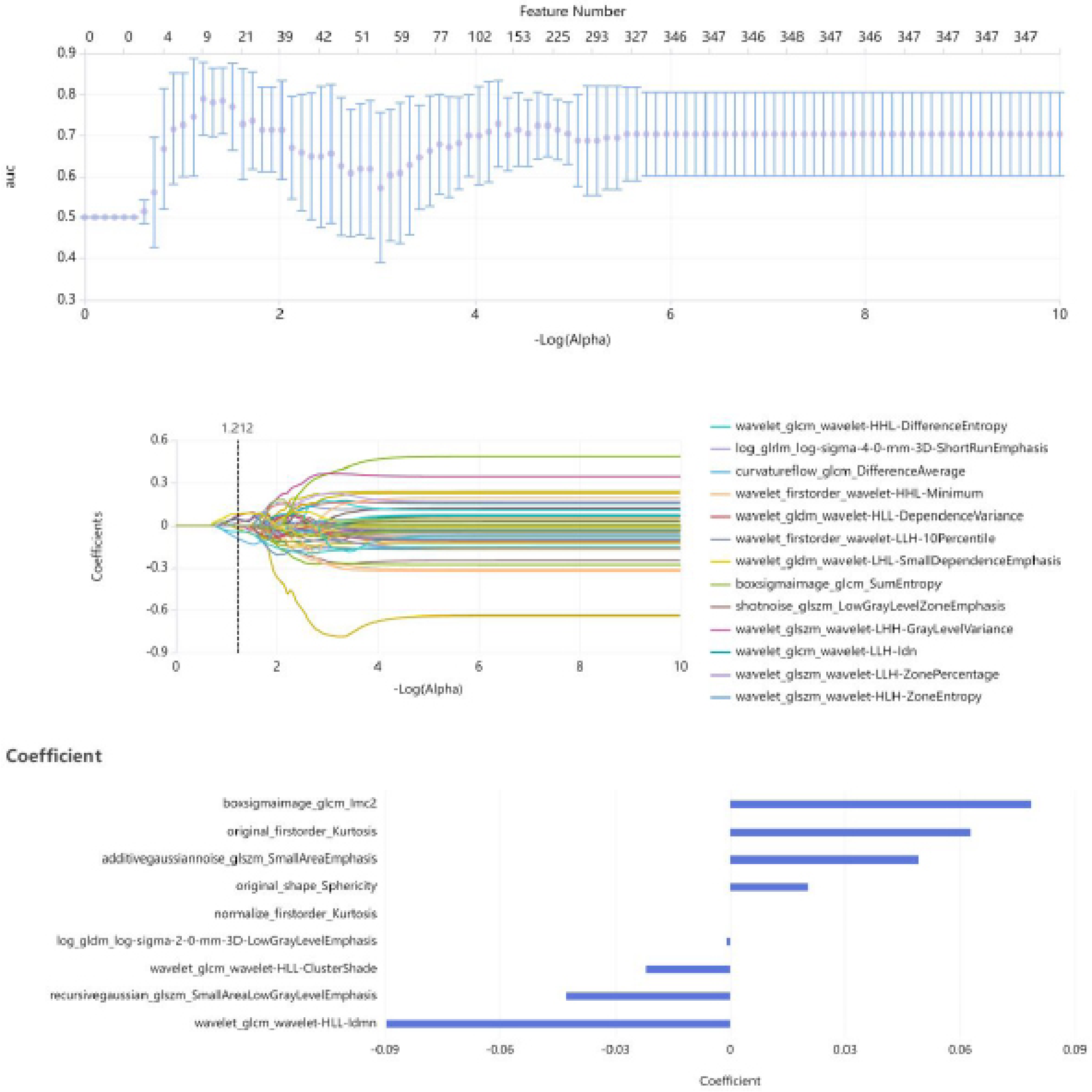

Fig. (S2). Radiomics feature extraction from T1 regions of interest.
The feature selection process involves preliminary elimination of irrelevant features through univariate analysis, followed by refined multivariate selection using Least Absolute Shrinkage and Selection Operator (LASSO) regression.

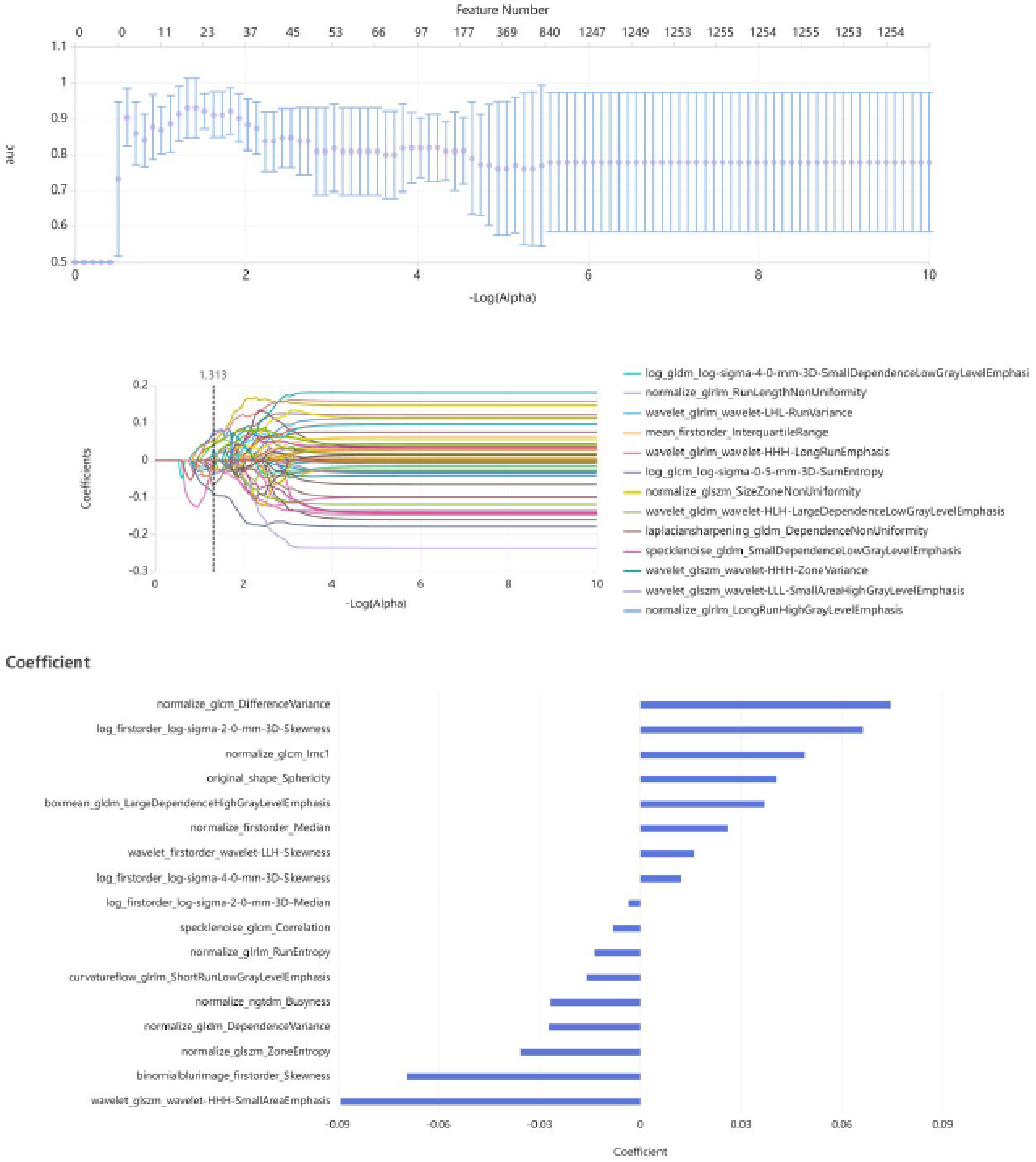

Fig. (S3). Radiomics feature extraction from T2 regions of interest.

The feature selection process involves preliminary elimination of irrelevant features through univariate analysis, followed by refined multivariate selection using Least Absolute Shrinkage and Selection Operator (LASSO) regression.

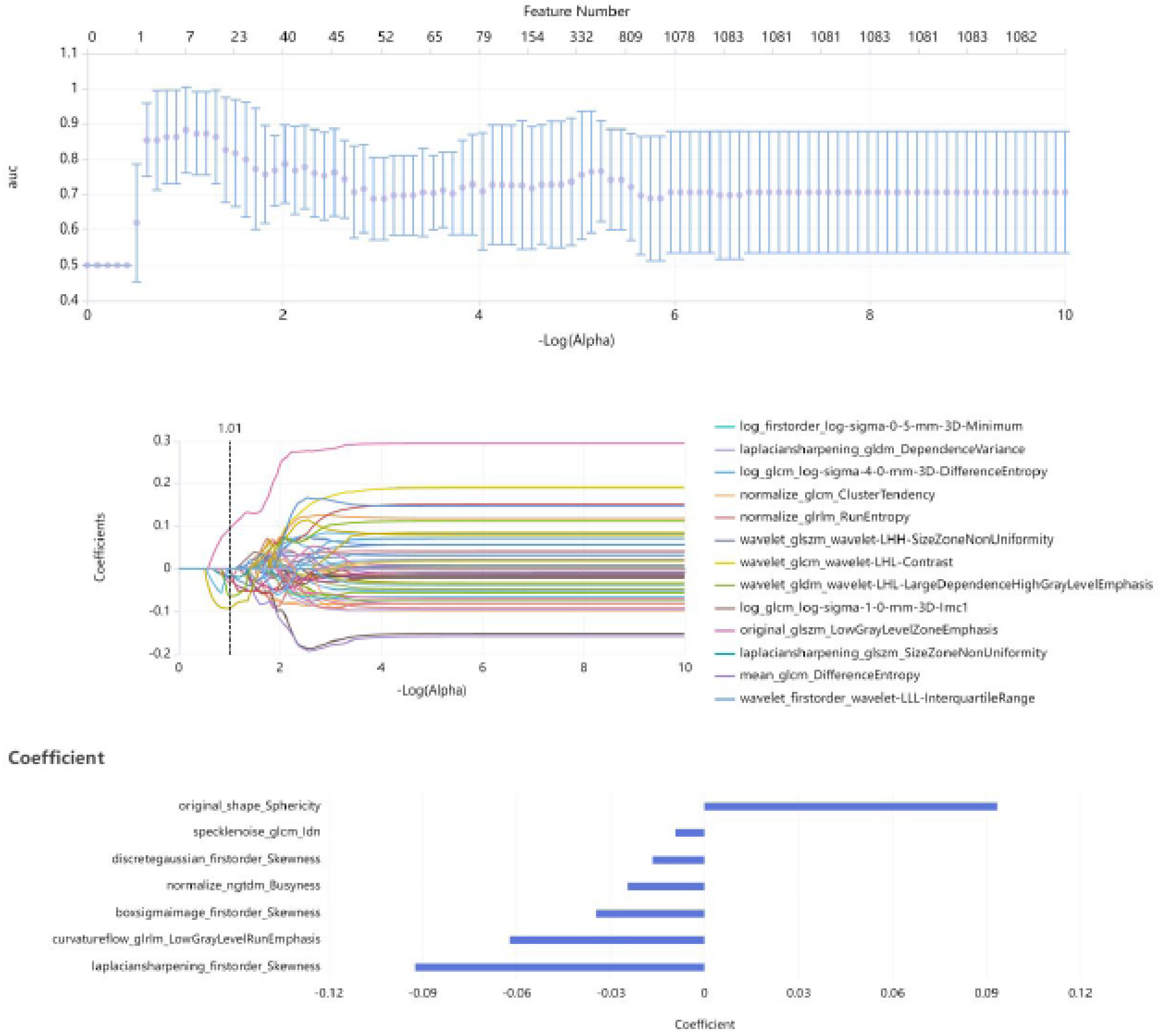

Fig. (S4). Radiomics feature extraction from T2-weighted fat-suppressed regions of interest.
The feature selection process involves preliminary elimination of irrelevant features through univariate analysis, followed by refined multivariate selection using Least Absolute Shrinkage and Selection Operator (LASSO) regression.

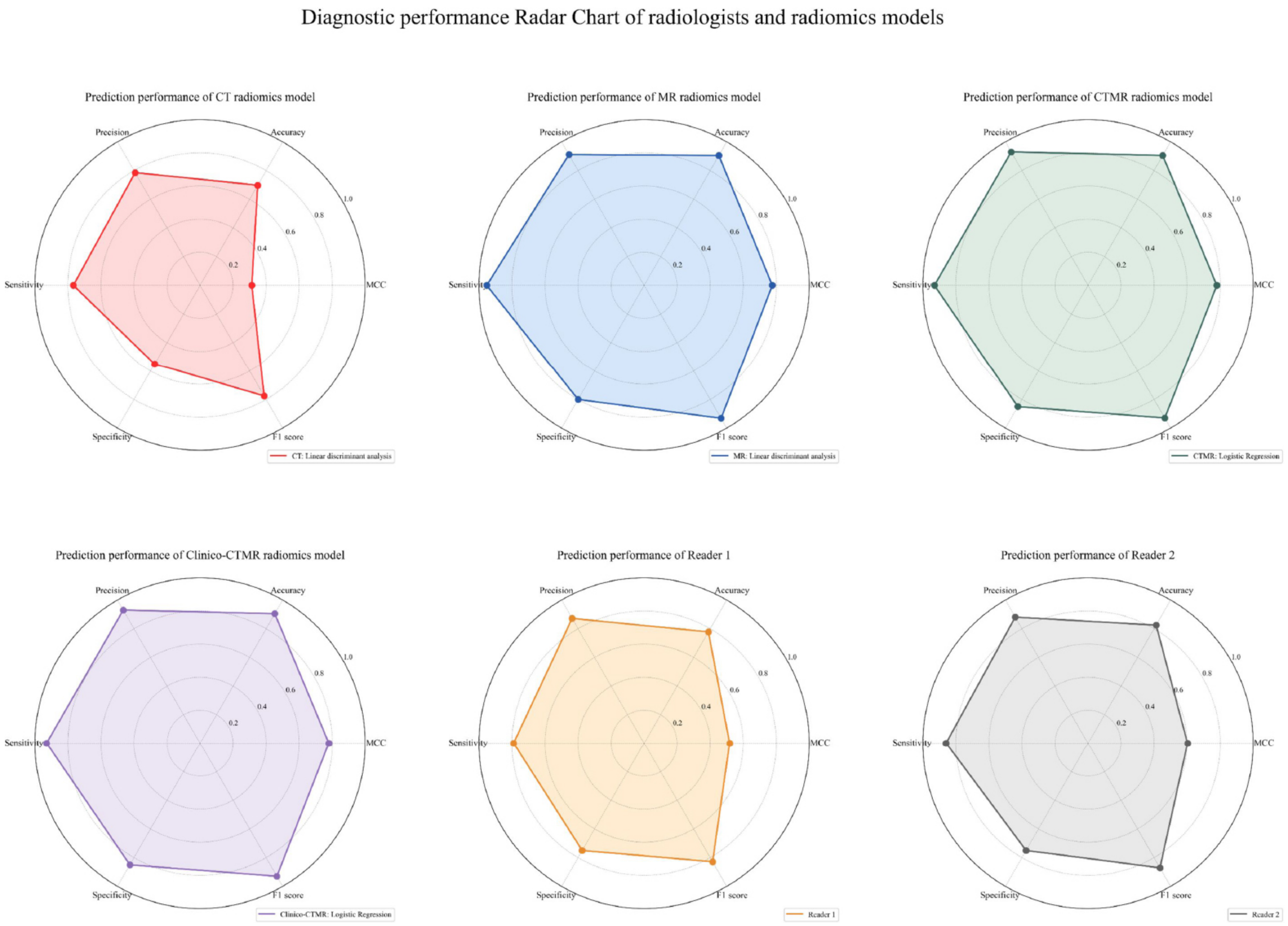

Fig. (S5). Diagnostic performance radar charts of different radiomics models and readers.

The radar charts visually depict the sensitivity, specificity, precision, accuracy, Matthews correlation coefficient and F1 score of the following: CT-based radiomics model, MR-based radiomics model, CTMR radiomics model, clinical-CTMR fusion model, and assessments by two independent radiologists.

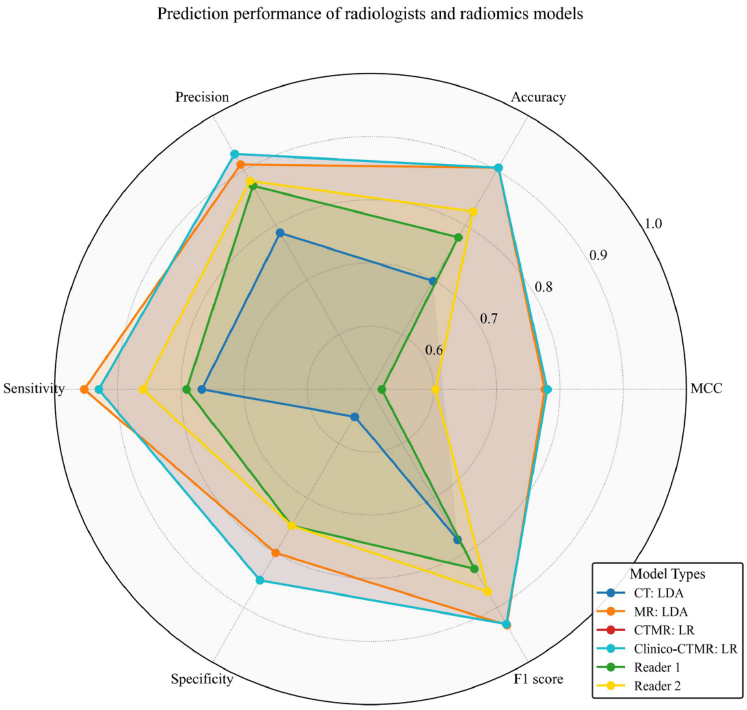

Fig. (S6). Diagnostic performance Radar Charts of different radiomics models and readers.
The combined radar chart illustrates sensitivity, specificity, precision, accuracy, Matthews correlation coefficient and F1 score of the following: CT-based radiomics model, MR-based radiomics model, CTMR radiomics model, clinical-CTMR fusion model, and assessments by two independent radiologists.

© 2025 The Author(s). Published by Bentham Science Publisher.

This is an open access article distributed under the terms of the Creative Commons Attribution 4.0 International Public License (CC-BY 4.0), a copy of which is available at: https://creativecommons.org/licenses/by/4.0/legalcode. This license permits unrestricted use, distribution, and reproduction in any medium, provided the original author and source are credited.
